# Sequencing, *De Novo* Assembly and Annotation of the Colorado Potato Beetle, *Leptinotarsa decemlineata*, Transcriptome

**DOI:** 10.1371/journal.pone.0086012

**Published:** 2014-01-23

**Authors:** Abhishek Kumar, Leonardo Congiu, Leena Lindström, Saija Piiroinen, Michele Vidotto, Alessandro Grapputo

**Affiliations:** 1 Department of Biology, University of Padova, Padova, Italy; 2 Department of Genetics & Molecular Biology in Botany, Institute of Botany, Christian-Albrechts-University at Kiel, Kiel, Germany; 3 Centre of Excellence in Biological Interactions Research, Department of Biological and Environmental Science, University of Jyväskylä, Jyväskylä, Finland; Hospital for Sick Children, Canada

## Abstract

**Background:**

The Colorado potato beetle (*Leptinotarsa decemlineata*) is a major pest and a serious threat to potato cultivation throughout the northern hemisphere. Despite its high importance for invasion biology, phenology and pest management, little is known about *L. decemlineata* from a genomic perspective. We subjected European *L. decemlineata* adult and larval transcriptome samples to 454-FLX massively-parallel DNA sequencing to characterize a basal set of genes from this species. We created a combined assembly of the adult and larval datasets including the publicly available midgut larval Roche 454 reads and provided basic annotation. We were particularly interested in diapause-specific genes and genes involved in pesticide and *Bacillus thuringiensis* (Bt) resistance.

**Results:**

Using 454-FLX pyrosequencing, we obtained a total of 898,048 reads which, together with the publicly available 804,056 midgut larval reads, were assembled into 121,912 contigs. We established a repository of genes of interest, with 101 out of the 108 diapause-specific genes described in *Drosophila montana*; and 621 contigs involved in insecticide resistance, including 221 CYP450, 45 GSTs, 13 catalases, 15 superoxide dismutases, 22 glutathione peroxidases, 194 esterases, 3 ADAM metalloproteases, 10 cadherins and 98 calmodulins. We found 460 putative miRNAs and we predicted a significant number of single nucleotide polymorphisms (29,205) and microsatellite loci (17,284).

**Conclusions:**

This report of the assembly and annotation of the transcriptome of *L. decemlineata* offers new insights into diapause-associated and insecticide-resistance-associated genes in this species and provides a foundation for comparative studies with other species of insects. The data will also open new avenues for researchers using *L. decemlineata* as a model species, and for pest management research. Our results provide the basis for performing future gene expression and functional analysis in *L. decemlineata* and improve our understanding of the biology of this invasive species at the molecular level.

## Introduction

The Colorado potato beetle, *Leptinotarsa decemlineata* (Say), is the major defoliator of potato throughout the northern hemisphere [1]–[5]. Both larvae and adults feed on potato plants causing damage to potato fields and financial losses to farmers [6]. The beetle is native to Mexico and south-eastern USA [7], where it lives on wild solanaceous species such as *Solanum rostratum* and *S. angustifolium* [8]. The shift to potato occurred sometime before 1850 in the US [9], when potato farming reached the distribution range of the beetle [2]. The beetle spread rapidly throughout the US reaching the east coast before 1880 [2], and was accidentally introduced to Europe (in France) in the 1920s. In 50 years it spread throughout Europe except for the UK and Scandinavia. Its current range covers 16 million km^2^ in North America, Europe and Asia. It is currently spreading further eastwards and also towards higher latitudes [9]–[12].

Several factors have contributed to the beetle's high success as an invader and a pest species: adaptation to the potato host, high fecundity, the ability to synchronize its life cycle through diapause, and a high capability to evolve resistance to insecticide [1], [13]. Diapause is a physiological state of dormancy that allows insects to escape unfavorable conditions, such as harsh winters or drought. The decision to enter diapause is mainly determined by day length, but is affected by food availability, temperature and moisture conditions [14]–[17]. Diapause is therefore a critical component of insect phenology [18]. More importantly, insecticide resistance can interact with diapause in insects, including *L. decemlineata* [19]–[22]. This gives diapause a central role in insect pest management [23], [24]. Developing a more comprehensive understanding of diapause and its influence on insect life-history traits will offer insights into other biological processes and help in planning new pest management strategies [25].

The first large-scale use of insecticides in agricultural crops was for the suppression of *L. decemlineata* [3]. Insecticides were very successful in controlling this serious pest until it developed widespread resistance to DDT in the mid-1950s [2]. In the laboratory conditions, the beetle has since developed resistance to at least 52 different compounds covering all the major pesticide classes, including pyrethroids, organophosphates, neonicotinoids [1], and, only in laboratory populations, also to *Bacillus thuringiensis* (Bt) [26], [27]. Despite this, insecticide compounds remain the most used and only efficient means of managing beetle populations. At the same time, there is growing concern over the evolution of resistance and the environmental consequences of increased dosages of insecticides [1], [28].

The beetle is important for both basic and applied biology – from invasive biology, through insect phenology to pest-species management. In fact, *L*. *decemlineata* has been included in the i5k insect genomes project (http://arthropodgenomes.org/wiki/Main_Page; http://www.ncbi.nlm.nih.gov/bioproject/PRJNA171749) in 2012 [29]. A first un–annotated draft of the genome has been made available while our manuscript was in its final preparation and therefore it could not be included in our analysis. Genetic investigations of *L. decemlineata* biology and its resistance to both chemical pesticides and Bt have relied on homology-based gene-by-gene cloning, on low throughput EST sequencing [18], [30], [31] and, more recently, the beetle larval midgut has been subject to 454 pyrosequencing [32].

Next-generation sequencing methods, such as 454 pyrosequencing, are cost-effective methods for the transcriptome characterization of insect species that lack a fully-sequenced genome [33]–[35]. The deeper sequencing coverage of the 454 method and an accurate base calling allow for *de-novo* transcriptome assembly and the characterization of genes without a reference genome. The massive number of expressed sequence tags obtained with this method facilitates the discovery and identification of new genes and the analysis of gene expression by providing a reference transcriptome for cDNA microarrays. It also facilitates the identification of such novel Type I genetic markers as microsatellites and SNPs for population genomic and quantitative traits locus (QTL) analyses [36], [37].

We used 454 FLX Titanium-based pyrosequencing to generate a substantial dataset of transcripts reads of the *L. decemlineata* transcriptome. Together with the publicly available midgut larval reads [32], we obtained 121,912 contigs, of which 41.15% were similar to known protein or nucleotide sequences. We performed the *in silico* identification of Type I genetic markers and characterized genes of interest for diapause, detoxification pathways and insecticide target proteins. We annotated the combined assembly, including 8,993 transcripts available at NCBI (June 2012). All data and assembly are available at http://www.bio.unipd.it/~grapputo/CPB-Webpage. Our results will provide the basis for performing future gene expression studies and functional analysis in *L. decemlineata* and improve our understanding of the biology of this invasive species at the molecular level.

## Results and Discussion

### Transcriptome assembly characteristics

Using the Roche 454 pyrosequencing method, we obtained 456,909 transcriptomic reads from adult beetles and 444,435 reads from beetle larvae for a total of 901,344 reads corresponding to 64.23 Mbp. After a cleaning step for removing adapters, low quality bases and contaminants (bacteria, viruses and potato sequences) we remained with 445,257 (97.45% of the original reads) and 442,791 (99.63%) reads for adults and larvae, respectively. These reads were combined with the publicly available 839,061 Roche 454 midgut larval reads [38], which, after cleaning as describe above, produced 804,056 (95.83%) reads. The total 1,702,104 reads from these three datasets were assembled into 117,848 contigs and 4,064 singletons for a total of 121,912 partial transcripts (**Table S1**) using the Mira 3.2 assembler [39]. Contig lengths varied from 41 to 7,034 bp with an average of 537 bp (**Figure S1 and Table S1**). Singletons ranged from 40 to 583 bp with an average length of 214 bp (**Tables 1** **and S1**). Contigs and singletons had an average GC content of 36% and 35%, respectively (**Tables S1)**. This was lower than the GC content (46%) of the red flour beetles (*Tribolium castaneum*) transcriptome suggesting that the genome of *L. decemlineata* could be very rich in AT as shown in other insects [40].

**Table 1:** Summary of assembly statistics.

**MIRA contigs (#)**	117,848
MIRA contigs on assembly (%)	96.67
length (Mb)	63.359063
average_length (bp)	537.634
median_length (bp)	426
min_length (bp)	41
max_length (bp)	7,034
GC_conten (%)	36.1
average quality (phred)	41.5986
MIRA average_coverage (reads/contig)	11.9784
N25 stats	25% of total sequence length is contained in the 9245 sequences > = 1,081 bp
N50 stats	50% of total sequence length is contained in the 30645 sequences > = 570 bp
N75 stats	75% of total sequence length is contained in the 63854 sequences > = 409 bp
MIRA average_coverage (no singlets) (bp/position)	4.02896
MIRA median_coverage (no singlets) (bp/position)	2.17
MIRA standard_dev coverage (no singlets) (bp/position)	6.2343
	
**MIRA Singletons (#)**	4,064
MIRA Singletons (%)	3.33
length (Mb)	0.87
average_length (bp)	214.044
median_length (bp)	205
min_length (bp)	41
max_length (bp)	538
GC_conten (%)	34.89
average quality (phred)	26.4683
N25 stats	25% of total sequence length is contained in the 521 sequences > = 369 bp
N50 stats	50% of total sequence length is contained in the 1,179 sequences > = 295 bp
N75 stats	75% of total sequence length is contained in the 2,065 sequences > = 203 bp

For simplicity we will not make further distinction between contigs and singletons and refer to both as contigs unless otherwise stated.

### BLAST analyses

**Figure 1:**
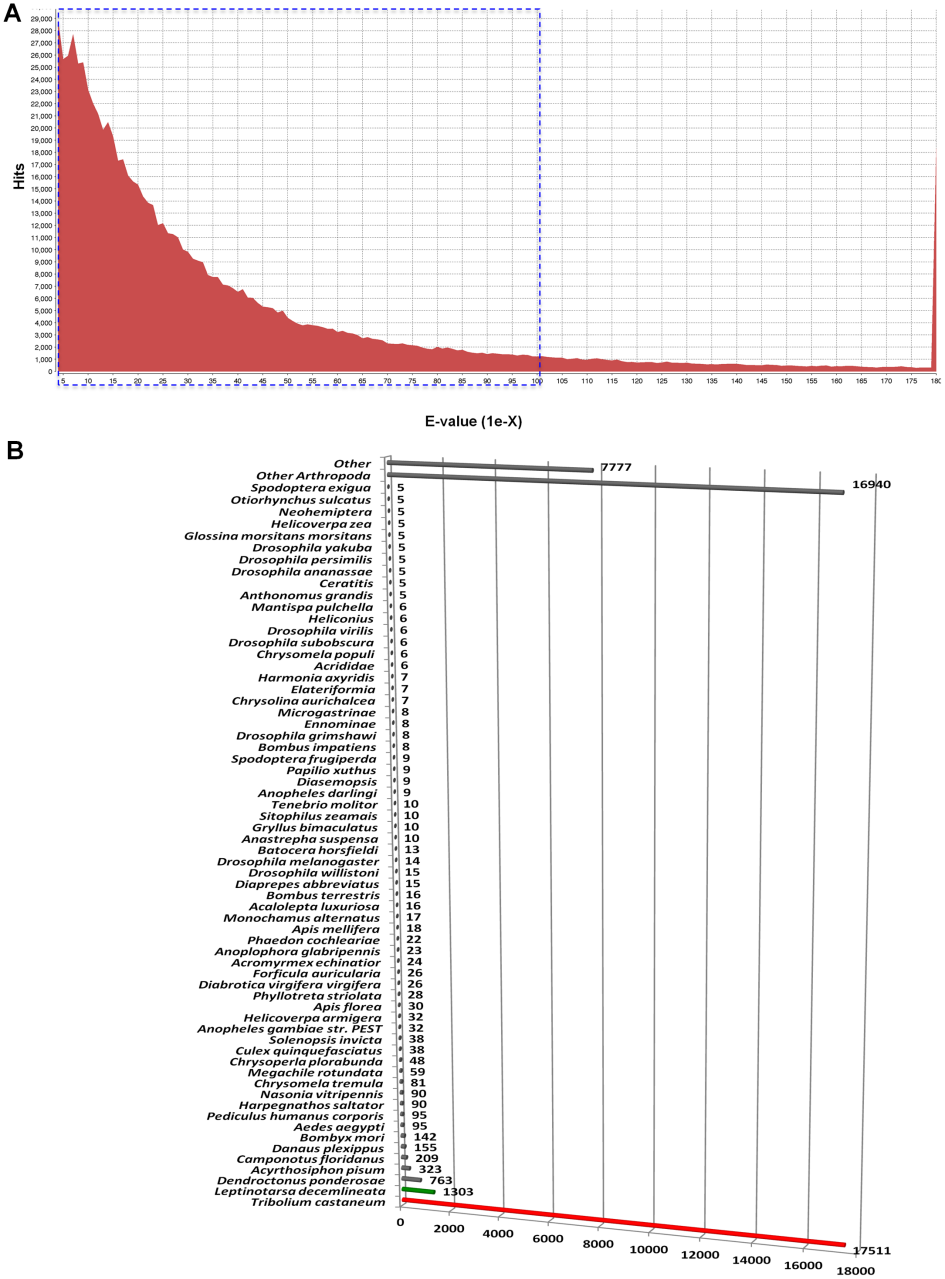
Summary of BLAST results for transcriptomic sequences vs Genbank non-redundant (nr) database. **A**. E-value distribution suggested that majority of hits ranged from 1e^−5^ to 1e^−100^ (blue dashed square). **B**. Taxonomic distribution of the top BLAST hits of *L. decemlineata* contigs. Majority of fractions belongs to insects with the beetles *T. castaneum* (red bar) and *L. decemlineata* (green bar) as the two top species. The low number of hits to *L. decemlineata* was due to the low coverage of this beetle in the current nr database.

Of 121,912 *L. decemlineata* contigs, 41.15% showed significant similarity (E value<1e^−3^) to proteins in the GenBank non-redundant (nr) database. Although we used an E-value threshold of <1e^−3^ for this analysis, the majority of matches were below this threshold, i.e. between 1e^−4^ and 1e^−180^ (**Figure 1A**). As we expected, the major fraction of sequences with hits in GenBank matched insect proteins (79.23%) (**Figure 1B**). Within insects, *T. castaneum* had the highest share of matches with 37.8% (**Figure 1B**). In contrast *L. decemlineata* had only 2.81% of the hits, confirming the low amount of annotated genomic information available on this species.

### GO ontology assignment

Functional annotation is an essential requirement for understanding the transcriptomic data of non-model organisms. Gene Ontology (GO) facilitates the functional characterization of genes, transcripts and proteins of many organisms in terms of cellular components, biological processes and molecular functions in a species-independent fashion [41]. Currently, this approach is a standard method for corroborating overall annotation of 454-sequenced transcriptome data [42]–[46]. We have used this method for the functional annotation of *L. decemlineata* transcripts using the Blast2GO suite [41].

**Figure 2:**
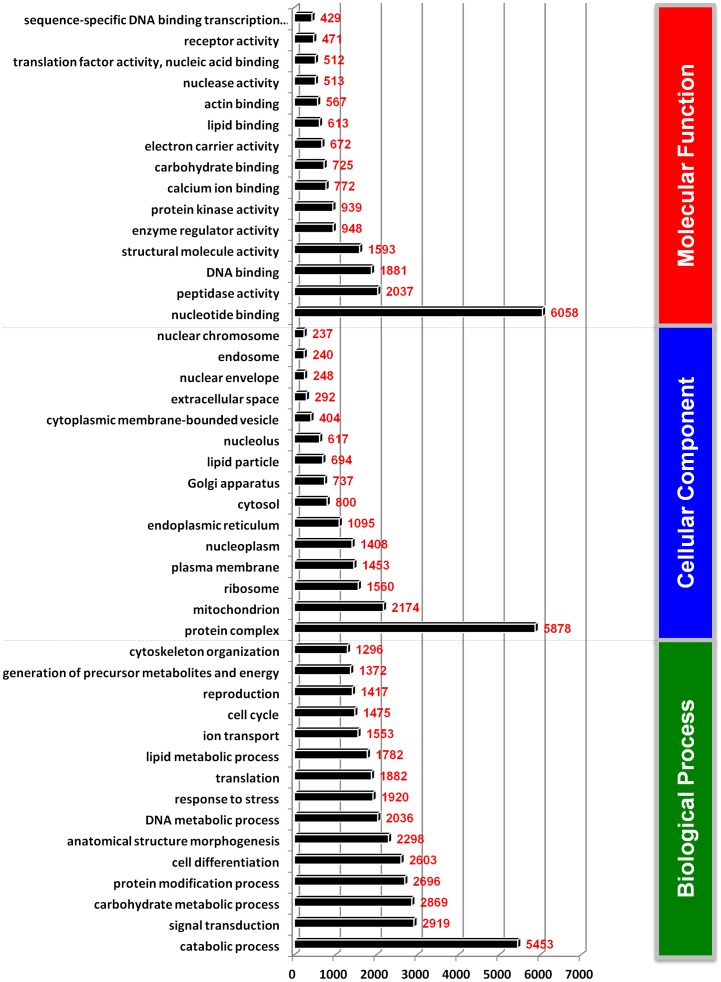
Description of the three categories of Gene Ontology (GO) terms for the transcriptomic sequences of *L. decemlineata*. The top 15 GO terms of each category are shown. A detailed summary is listed in **Table S4**.

The derived *L. decemlineata* transcripts were assigned to three functional groups based on GO terminology: biological process, molecular function and cellular component (**Table S2**). We traced 42,208 contigs to biological process terms (**Figure 2**) with the following five top categories: catabolic process (5,453) signal transduction (2,919) carbohydrate metabolic process (2,869), protein modification process (2,696) and cell differentiation (2,603). Similarly, 18,738 contigs were assigned to cellular component terms (**Figure 2**) with the five top components being: protein complex (5,878), mitochondrion (2,174), ribosome (1,560), plasma membrane (1,453) and nucleoplasm (1,408). Finally, 20,438 contigs were linked to molecular function terms (**Figure 2**) with nucleotide binding (6,058), peptidase activity (2,037), DNA binding (1,881), structural molecule activity (1,593) and enzyme regulator activity (948), being the five top categories. All GO terms in these three categories are listed in **Table S3.** Further, we assigned 667 enzymatic codes (EC) to 16,656 contigs (**Table S3**) encompassing all six groups, EC1–EC6, with the highest transcript numbers assigned to EC2 and the lowest to EC5 (**Table S3**). A total of 539 transcripts were assigned more than one enzymatic code.

The annotated contigs are provided on our group website (http://www.bio.unipd.it/~grapputo/CPB-Webpage/).

### Enzymatic pathway analyses

**Figure 3:**
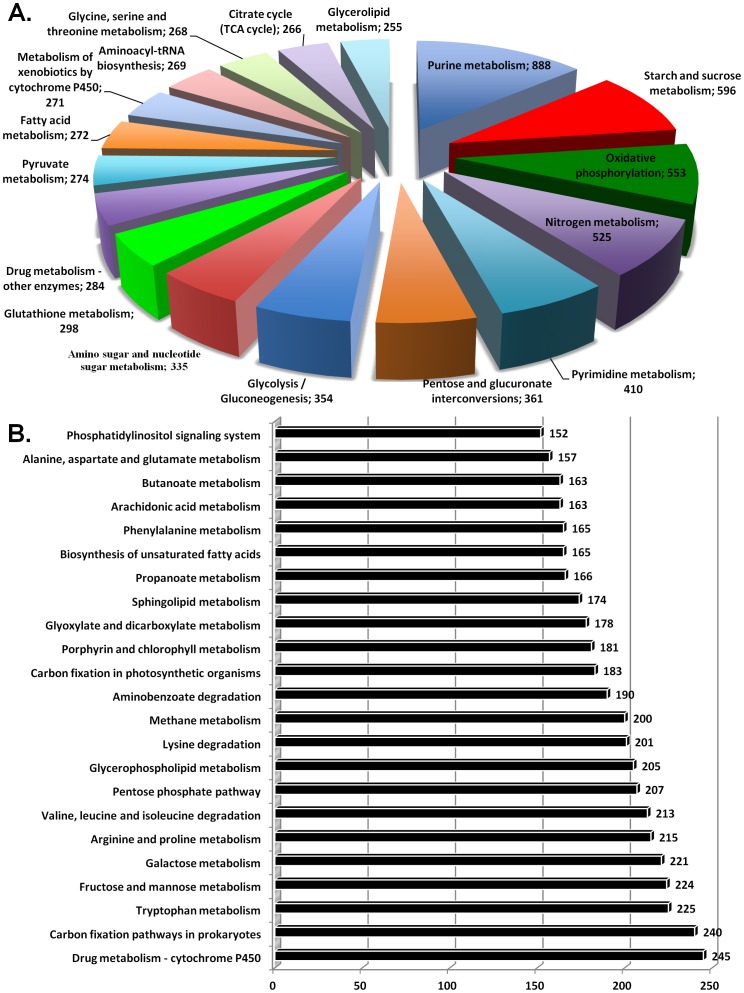
Summary of enzymatic KEGG pathways. **A**. KEGG pathways comprising more than 250 transcriptomic sequences. **B**. KEGG pathways comprising transcriptomic sequences between 150 and 250. A detailed summary is listed in **Table S5**.

We mapped 16,656 contigs to 663 enzymes of 134 different reference canonical pathways in the Kyoto Encyclopedia of Genes and Genomes (KEGG) (**Table S4**). The coverage of transcriptomic sequences per pathway ranged from 1 to 888; whereas the coverage of these sequences per enzyme ranged from 1 to 224. There were 17 pathways covered by more than 250 sequences (**Figure 3A**). The top five were: purine metabolism (888 sequences; KEGG map00230), starch and sucrose metabolism (596; map00500), oxidative phosphorylation (553; map00190), Nitrogen metabolism (525; map00910) and pyrimidine metabolism (410; map00240). A further 23 KEGG pathways had a coverage range of between 150 and 250 putative enzymatic sequences (**Figure 3B**). The ratio of contig to singleton sequences in KEGG pathways was approximately 3.4∶1. Similar results for KEGG pathways have been observed for other insect transcriptomes [43]–[46].

### Identification of putative microRNAs

MicroRNAs (miRNAs) are small non-coding RNAs that have significant roles in the regulation of gene and protein expression in various biological processes [47], [48]. In order to identify putative novel microRNAs in the transcriptome of *L. decemlineata*, we scanned all known metazoan microRNA sequences from a miRNAs database (miRBase - Release 20, June 2013) [49]. We identified 460 putative miRNA as summarized in **Table S5.**

### Comparison of adult and larval transcriptomes

Since each read was labeled with the library of origin before the assembly, we were able to identify those contigs formed by reads from either the adult or the larval transcriptome and those from either one of the two larval transcriptomes. Out of 212,912 contigs obtained from the combined assembly of the three data sets, 43,050 contained reads from both adults and larvae while 19,811 and 59,051 contained reads from only adults and from only larvae (full larval [FL] data set + midgut larval [ML] data set). The comparison between the two larval transcriptomes allowed to identify contigs entirely composed by reads of the same library. These putative specific contigs were 18,470 for FL and 30,540 for ML.

### Genes of Interest

We searched for homologies between *L. decemlineata* sequences and insect model genomes, such as *Drosophila* and *Tribolium*, focusing on genes putatively involved in diapause, detoxification and insecticide resistance.

#### Diapause-associated genes

**Figure 4:**
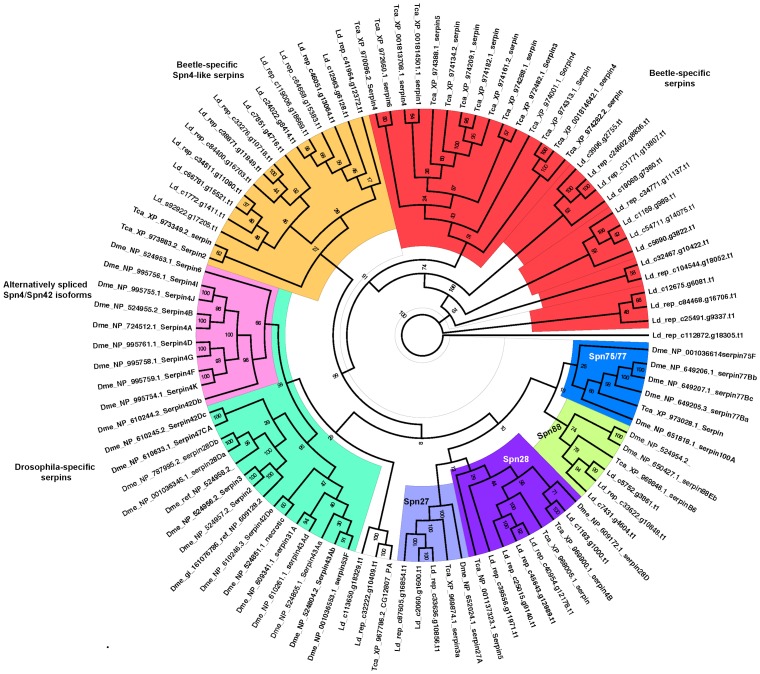
Bayesian phylogeny of serpins from *T. castaneum* and *D. melanogaster* genomic and *L. decemlineata* transcriptomic sequences. Several serpins are specific for *D. melanogaster* (green box) with many alternatively-spliced isoforms of Spn4 (synonym Spn42, pink box) whereas beetle specific Spn4-like serpins are marked in yellow. *L. decemlineata* has several tandemly-duplicated serpins (red box), as previously reported in *T. castaneum* on chromosome 8 [53]. The tree was based on the WAG +G+I protein model and run for 10,342,000 generations. Posterior probability values are indicated at the nodes. Sequences are named by a prefix of three letters indicating the species (Dme – *D. melanogaster*, Tca – *T. castaneum*, Ld – *L. decemlineata*) followed by either the accession ids and name in NCBI (for Dme and Tca) or contig name (for Ld).

We scanned for protein sequences encoded by eight transcripts previously reported to be down-regulated as beetles enter the diapause-maintenance phase of diapause development [18]. These transcripts are summarized in **Table S6**. To explore how many homologs are found in our transcriptome dataset in comparison to *Drosophila* and *Tribolium*, we used serpin as a test case. The serpin superfamily is characterized by a highly conserved domain of 35–50 kDa and these protease inhibitors play essential roles in the regulation of proteolytic cascades [50], [51]. About 30 different serpins have been previously identified in other insects with some alternatively-spliced isoforms [52]–[54]. We report 41 contigs with homologous to serpins from the *L. decemlineata* transcriptome, which include most of the serpins known in insects as summarized by a Bayesian phylogenetic tree (**Figure 4**). Several serpins are *Drosophila*-specific including alternatively-spliced isoforms of Spn4/Spn42 (**Figure 4**) [52]–[54]. Similarly, as the phylogenetic tree shows, *L. decemlineata* presents several serpins arose likely by several duplication events (red in **Figure 4**), as previously reported in *T. castaneum* on chromosome 8 [53], suggesting that this duplication event could be common to all Cucujiformia or even all Coleoptera.

A recent study on *Drosophila montana* unraveled 108 genes that are instrumental in photoperiodic reproductive diapause [55]. Upon scanning these 108 genes, 101 were found in the beetle transcriptome spanning different functional categories (**Table 2**) with various homologs as listed in **Table S7**. The seven diapause candidate genes missing in this combined assembly were: disco, couch potato (cpo), CrebB17A, inaF, narrow abdomen (na), nnchung (nan) and timeless. **Table S7** also shows the results of the identification of dataset specific transcripts indicating that all these 101 transcripts involved in reproductive diapause in drosophila were expressed in both larvae and adult beetles. With our data we could not investigate differential expression of these transcripts in the different stages and therefore further studies are needed to assess if these genes have a role in the beetle diapause.

**Table 2:** Summary of putative genes involved in diapause in comparison to *D. montana.*

Function	*D. montana*	*L. decemlineata*
Circadian rhythm	26	23
Cold tolerance	02	02
Courtship behavior	14	13
Diapause	07	05
Heat tolerance	27	27
Housekeeping gene	07	07
Phototransduction	25	24
**Total**	**108**	**101**

#### Putative transcripts involved in insecticide resistance

Since the first insecticide applications against the Colorado potato beetle in the 1950s, this pest has developed resistance to all the major classes of chemical insecticides (reviewed in [1], [28]). Resistance mechanisms can be highly diverse even within a small geographic area [56], involving a variety of different genes including cytochrome P450 monoxygenases (CYPs), glutathione S-transferases (GSTs), superoxide dismutases (SODs), catalases (CATs), ADAM metalloproteases (ADAMs), cadherins (CADHs), Calmodulins (CALMs), glutathione peroxidases (GPXs), esterases and ascorbate peroxidases. Insect CYPs are also important in metabolizing plant secondary metabolites [57] and Zhang et al. [58] found 38 up-regulated CYPs in Colorado potato beetles when the insects were fed on Solanaceae.

We have created a catalogue of genes putatively involved in insecticide resistance in *L. decemlineata* from the transcriptome assembled here using BLAST homology searches [59] at E-values lower than 1e^−3^ and listed in **Table 3**. The contigs encoding these genes are not necessarily complete ORFs and therefore it is likely that several contigs correspond to the same gene. From our dataset, it is difficult to discriminate between alleles of the same gene from recent duplication events as we sequenced a pool of many individuals. Therefore the number of genes per gene family could have been overestimated. However, the number of genes reported in **Table 4** is smaller than the number reported in the mosquito *Culex pipiens quinquefasciatus* [60].

**Table 3:** Summary of genes that induce insecticide resistance in *L. decemlineata*.

Gene	Contigs in Ld_Assembly	Adult	Larva	FL only	ML only
**Catalases (CAT)**	13	0	9	5	1
**Glutathione peroxidases (GPX)**	22	3	8	3	2
**Glutathione S-transferases (GSTs)**	45	2	11	4	4
**ADAM metalloprotease (ADAM)**	3	1	0	0	0
**Cadherin (Cadh)**	10	1	5	1	4
**Calmodulin (CalM)**	98	13	30	8	14
**Cytochrome P450 monoxygenases (CYPs)**	221	24	77	20	44
**Esterase**	194	17	111	5	89
**Superoxide dismutases (SOD)**	28	4	4	2	2

Genes were scanned by BLAST [59] at E-value lower than 1e^−3^. The table also shows the number of contigs identified as library specific for each gene.

FL – full larva; ML – midgut-larva.

**Table 4:** Summary of top Pfam protein domains in *L. decemlineata* transcriptome.

Accession id	Pfam domain name	Domain description	# Occurrence
PF00096	zf-C2H2	Zinc finger C2H2 type	878
PF00400	WD40	WD domain G-beta repeat	850
PF00560	LRR_1	Leucine Rich Repeat	556
PF07719	TPR_2	Tetratricopeptide repeat	516
PF00023	Ank	Ankyrin repeat	465
PF00515	TPR_1	Tetratricopeptide repeat	419
PF00036	efhand	EF hand	412
PF00069	Pkinase	Protein kinase domain	324
PF02985	HEAT	HEAT repeat	279
PF00076	RRM_1	RNA recognition motif. (a.k.a. RRM RBD or RNP domain)	267
PF07714	Pkinase_Tyr	Protein tyrosine kinase	251
PF07679	I-set	Immunoglobulin I-set domain	220
PF00112	Peptidase_C1	Papain family cysteine protease	177
PF00435	Spectrin	Spectrin repeat	175
PF00005	ABC_tran	ABC transporter	169
PF00379	Chitin_bind_4	Insect cuticle protein	164
PF01607	CBM_14	Chitin binding Peritrophin-A domain	157
PF08246	Inhibitor_I29	Cathepsin propeptide inhibitor domain (I29)	154
PF00135	COesterase	Carboxylesterase	148
PF00067	p450	Cytochrome P450	145
PF00153	Mito_carr	Mitochondrial carrier protein	143
PF00047	ig	Immunoglobulin domain	140
PF00232	Glyco_hydro_1	Glycosyl hydrolase family 1	134
PF00514	Arm	Armadillo/beta-catenin-like repeat	128
PF00106	adh_short	short chain dehydrogenase	126
PF00271	Helicase_C	Helicase conserved C-terminal domain	124
PF00004	AAA	ATPase family associated with various cellular activities (AAA)	114
PF00018	SH3_1	SH3 domain	110
PF00071	Ras	Ras family	108
PF07690	MFS_1	Major Facilitator Superfamily	104

Pfam protein domains were scanned at E-value*<*1e^−3^.

**Figure 5:**
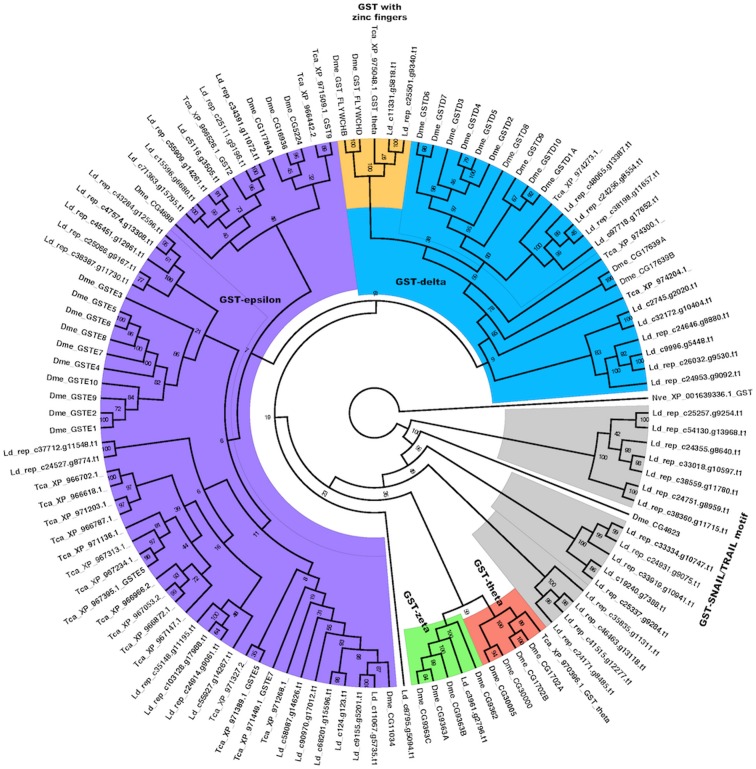
Bayesian phylogeny of GSTs from *T. castaneum*, and *D. melanogaster* genomic and *L. decemlineata* transcriptomic sequences. The tree was based on the WAG +G+I protein model and run for 9,248,000 generations. Posterior probability values are marked at the nodes. Sequences are named by a prefix of three letters indicating the species (Dme – *D. melanogaster*, Tca – *T. castaneum*, Lde – *L. decemlineata*) followed by either the accession ids and name in NCBI (for Dme and Tca) or contig name (for Ld). A GST from *N. vectensis* (Nve) was used as outgroup.

GSTs are a diverse family of enzymes that play a central role in insecticide resistance in insects. GSTs can metabolize insecticides by facilitating their reductive dehydrochlorination or by conjugation reactions with reduced glutathione to produce water-soluble metabolites that are more readily excreted [61], [62]. One or more GSTs have been implicated in resistance to organophosphates in *Musca domestica*, to organochlorine 1,1,1-trichloro-2,2-bis(*p*-chlorophenyl)-ethane (DDT) in *D. melanogaster* and to pyrethroids in *Nilaparvata lugens* [63]. We examined the status of GSTs in our assembled transcriptome and compared to *T. castaneum* and *D. melanogaster.* We found 45 contigs homologous to GSTs in *L. decemlineata* transcriptome sequences and their number is comparable to other insects with the expansion of epsilon and delta sub-groups as illustrated in the Bayesian phylogenetic tree (**Figure 5**). GSTZs are found in many eukaryotic species, including insects [64]. They are implicated in the detoxification of xenobiotics containing chloride in the silkmoth, *Bombyx mori* [62], [65]. We found one contig (Ld_c3961) homologous to the zeta-class glutathione S-transferases (GSTZs). This class of GST has not been observed in the red flour beetles and in the mosquito *C. p. quinquefasciatus* [60].

*L. decemlineata* laboratory populations have shown resistance to crystal (Cry) proteins derived from *Bacillus thuringiensis* (Bt) [26], [27]. Transgenic crops producing toxins and Cry proteins of Bt continue to be widely used in insect pest management. Mechanisms of resistance are different in different species and resistance to Bt has been subject of several studies also in the beetle [27], [66], [67]. On *L. decemlineata*, it has been shown that ADAM metalloprotease serves as receptor for Cry3Aa toxin [68]. Cry3Aa toxin specifically binds to calmodulin (CalM) in a calcium-independent manner [69] and also to the toxin-binding fragments of cadherin [70]. We have taken in consideration these three genes in our analyses and using scientific literature, we built a catalog of those genes known to be involved in resistance to Bt, including several transcripts recently isolated from *Diabrotica virgifera* showing responses to the Bt toxin Cry3Bb1 [71]. We traced these transcripts in our assembly and found the majority of them present (**Table 3**) with contigs Ld_c74929, Ld_rep_c32791 and Ld_c240 the closest hits for ADAM, CalM and cadherin, respectively.

**Figure 6:**
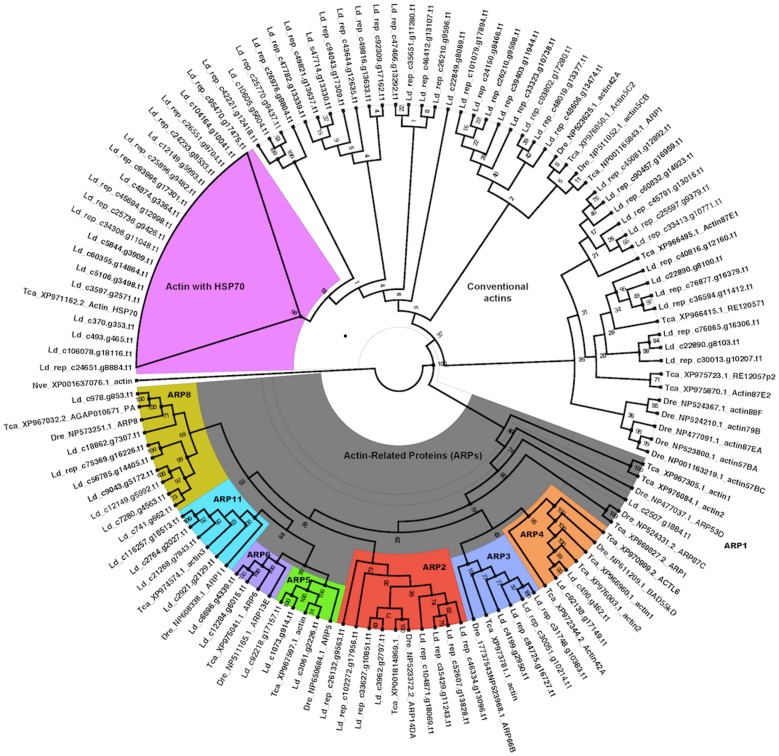
Bayesian phylogeny of actin and actin-related protein (ARP) from *T. castaneum*, and *D. melanogaster* genomic and *L.* decemlineata transcriptome sequences. The tree was based on the BLOSUM+G protein model and run for 10,927,000 generations. Posterior probability values are marked at the nodes. Sequences are named by a prefix of three letters indicating the species (Dme – *D. melanogaster*, Tca – *T. castaneum*, Ld – *L. decemlineata*) followed by either the accession ids and name in NCBI (for Dme and Tca) or contig name (for Ld). An actin from *N. vectensis* (Nve) was used as outgroup.

We selected actin, among the genes overexpressed in responsive *D. virgifera* to Bt (from **Table S8**), and examined how many actin homologs are expressed in the beetle transcriptome. We scanned actin transcripts in *L. decemlineata* and compared them with known actins in *T. castaneum* and *D. melanogaster*. Actin is a major contractile protein found in all eukaryotic cells and constitutes 1–2% of the total cellular protein of eukaryotic genomes [72], [73]. Several actin-like proteins, known as actin-related proteins (ARPs), are also present in various eukaryotic organisms. There are at least eight different ARP sub-families conserved in insects with different physiological roles such as actin polymerization (ARP2-3), chromatic remodeling (ARP4-6 and ARP8) and dynein mobility (ARP1 and ARP10) [72], [73]. We identified several contigs homologous to the majority of conventional actins and the actin-related proteins (ARPs) of *T. castaneum* and *D. melanogaster* as depicted by the Bayesian phylogenetic tree (**Figure 6** **)**.

In summary, we scanned the combined assembly of the adult and larval transcriptome of *L. decemlineata* for genes of interest. We found a comparable number of genes to, and which were conserved in, two other insects models, *T. castaneum* and *D. melanogaster*. We also carried out Bayesian phylogenetic tree for three representative genes from this dataset. The number of hits was generally higher for the larva than the adult transcriptome as it was higher for the ML than the FL dataset. This could be due to the higher number of reads sequenced for the midgut larval transcriptome which generated 1.65 times more contigs than ours datasets. An alternative hypothesis would be that most of Bt target receptors are specifically expressed in the midgut.

### Status of frequently-occurring eukaryotic Pfam protein domains

Protein domains are the building blocks of proteins as well as their evolutionary conserved units. The Pfam database is a large collection of multiple sequence alignments covering approximately 13,000 protein families [74]. The curated protein domains in Pfam have been used extensively in the annotation of new genomes and transcriptomes. We traced full Pfam domains in the *L. decemlineata* transcriptome using the CLC Genomics Workbench 6.05 [75]. We found a total of 8,927 transcripts associated with all eukaryotic protein pfam domains with E-values below 1e^−3^. The zinc finger type C2H2 topped these top domains with a total of 878 hits (**Table 4**). Zinc finger domains are relatively small protein motifs which contain multiple finger-like protrusions that make tandem contacts with their target molecule such as DNA, RNA, protein or lipid and they regulate gene expression in different eukaryotes during various processes, such as photoreceptor cell specification and differentiation [76], [77] Eye development [78] and larval-pupal metamorphosis [79] in beetles are governed by these zinc finger proteins. We detected 850 WD40 domains, involved in signal transduction, transcription regulation, cell cycle control, autophagy and apoptosis [80].

We found two tetratricopeptide-repeat-carrying domains, TPR_1 and TPT_2, in 419 and 516 transcripts, respectively. These motifs are involved in protein—protein interactions [81], [82].

We also found 145 cytochrome P450 domains in *L. decemlineata* transcriptomic sequences at E-values below 1e^−3^. Cytochrome P450s play an important role in the metabolism of xenobiotics and there is a known correlation between induced levels of these P450 genes and resistance to synthetic insecticides [83].

Gag proteins mediate the telomer-specific transposition of retrotransposons for telomer maintenance in *Drosophila* [84], [85] and a similar role of gag proteins was expected in *L. decemlineata*. We found 108 domains associated with reverse-transcriptase activity, which argues for transposable activities in this beetle. Furthermore, we found 159 transcripts coupled with the trypsin domain, a classical serine protease domain.

### Molecular markers

We identified 29,205 putative single nucleotide polymorphisms (SNPs) in 8,181 sequences (**Table 5** and **Table S9**). This number is likely an underestimate of the number of SNPs in this species as we collected samples only in Europe where *L. decemlineata* was subject to a founder event during its invasion of the Eurasian continent with a substantial reduction in genetic diversity [10]. Among these SNPs, 18.882 were transitions (Ts) and 10,383 were transversions (Tv) with a Ts to Tv ratio of 1.8∶1. We also predicted 17,284 single sequence repeats (SSRs or microsatellites). The majority of these microsatellites were di-nucleotide repeats (n = 13,473) while 3,294 were tri-nucleotide, 416 tetra-nucleotide, and 101 were penta-nucleotide repeats (**Table 6** and **Table S10**). These molecular markers, still to be verified by primer design and PCR amplification, could establish a platform for the research community to study the ecology, biology and genetics of *L. decemlineata* [86]. These markers, being EST-linked, may also provide a suitable tool to identify footprints of selective pressures due to natural or anthropogenic stress.

**Table 5:** Summary of putative SNPs in *L. decemlineata* transcriptomic sequences.

SNP types	Number
**Transition**	
A-G	9,515
C-T	9,306
**Transversion**	
A-C	2,394
A-T	4,049
C-G	1,567
G-T	2,368
T-R	5
G-R	1
**Total**	29,205

R – purine (G or A).

**Table 6:** Summary of microsatellite loci predicted in the *L. decemlineata* transcriptome.

	Nucleotides in repeats				
Number of repeats	Di-	Tri-	Tetra-	Penta-	
4	11,603	2,368	306	54	
5	1,264	577	70	14	
6	329	220	23	8	
7	144	72	10	2	
8	64	31	1	1	
9	33	11	0	5	
10	12	9	1	2	
11	5	3	0	1	
12	7	0	3	0	
13	5	0	0	2	
14	2	1	1	1	
15	0	0	0	2	
16	1	0	0	1	
17	1	0	0	0	
18	0	0	0	1	
19	2	0	0	2	
20	0	0	0	0	
21	0	0	0	1	
22	0	0	0	0	
23	0	0	1	0	
24	0	0	0	1	
25	0	0	0	0	
26	0	0	0	0	
27	0	0	0	0	
28	0	0	0	1	
30	0	1	0	1	
31	0	0	0	0	
32	0	0	0	0	
33	0	1	0	0	
36	0	0	0	0	
38	0	0	0	0	
41	0	0	0	1	
141	1	0	0	0	
**Total**	**13,473**	**3,294**	**416**	**101**	**17,284**

## Conclusions

We have established a new genetic resource for *L. decemlineata*, a species of high importance in the field of invasion biology. The major results of this study are: (1) a general annotation of *L. decemlineata* expressed genes; (2) the identification of a significant number of enzymatic pathways from these transcripts; (3) a catalog of putative SNPs and microsatellite markers which, upon validation, could facilitate the identification of polymorphisms within and between *L. decemlineata* populations; and (4) the characterization of genes of interest: those involved in diapause, detoxification and insecticide resistance. *L. decemlineata* is an important pest beetle species and is commonly used for studying plant-herbivore interactions and resistance to insecticides. A transcriptome assembly is, therefore of great importance to this community. The new genetic resource and putative miRNA candidates established by our study provide new insights into the biology of *L. decemlineata*.

## Materials and Methods

**Figure 7:**
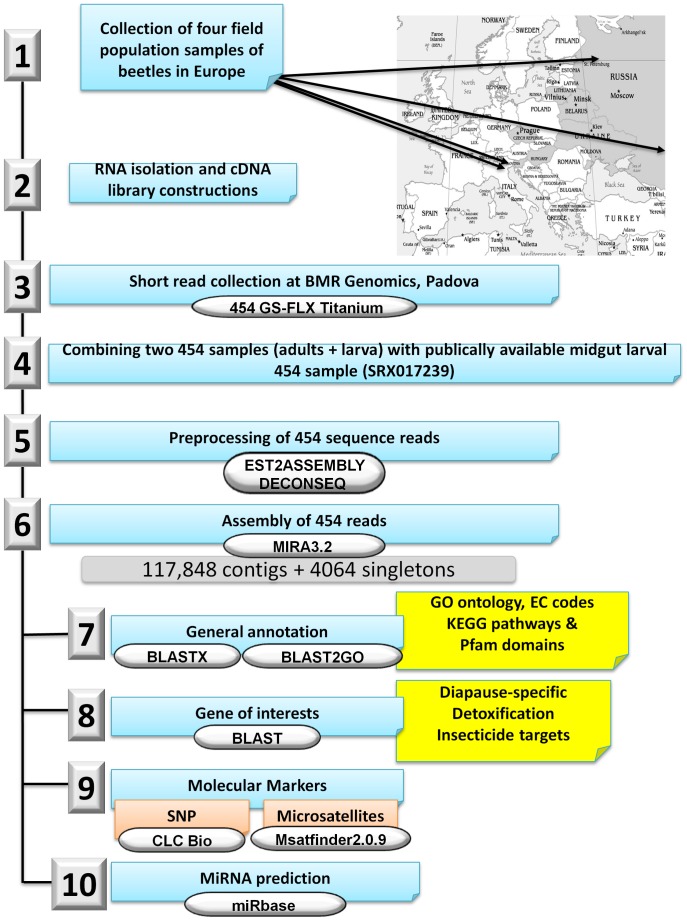
Overall approach to *L. decemlineata* 454 transcriptome analysis. Analysis steps are numbered and the software and tools used in each step are shown.

We have summarized our entire *L. decemlineata* transcriptome analysis approach in **Figure 7** **.**

### Beetle samples within Europe

Beetles were collected from four field populations, two in north-eastern Italy (Camposampiero 45 33 39.72 – 11 56 52.08 and Montello 45 47 49.10 – 12 07 17.85) and two in Russia (one near Petroskoy 61 48 28.81 – 34 7 51 and one near Ufa 54 47 05.26 – 55 57 57.69). No official permits were required to collect the beetles. Permission was granted by land owners to access the fields, which were not in protected areas. No endangered or protected species were involved in the project. Permits were obtained to take the beetles into Finland (Evira DNr: 4140/0614/2008). Beetles from Russia and Camposampiero were reared in the Finnish laboratory (Evira permit DNr: 3861/541/2007) on Van Gogh potato plants and at a constant 23°C. Beetles from Montello were reared in the laboratory in Padova (no permits are required to rear beetles in Italy) on the Monalisa potato and at a constant 24°C. Beetles were mated in the laboratory and larvae were grown on potato plants until adults emerged. Half of the samples from St. Petersburg and Camposampiero were grown under short-day length conditions (12L∶12D) to induce diapause and the other half under long-day length (18L∶6D). Twenty-three adults, 12 females and 11 males (6 individuals per population except for Montello, 5 individuals), were collected at intervals – from adult emergence to diapause – and preserved either at -80°C or in RNA later at −20°C. A total of 142 larvae were also collected at different instar stages (1 to 4) from the four populations and preserved either at −80°C or in RNA later at -20°C. Ten of these larvae (first instar) from Montello were exposed to potato leaf dipped in a solution of 3 mg/ml of *Bacillus thuringiensis* var *tenebrionis* strain NB 176 sierotype H 8a8b crystal proteins (Novodor FC; Serbios).

### RNA isolation and cDNA library construction

RNA was extracted from 23 adults and 142 larvae of *L. decemlineata* using the RNeasy Mini Kit (Qiagen) following the manufacturer's protocol for animal tissues. The RNA was extracted from 20 mg of tissue from the head, thorax or abdomen of adult beetles, from the head of 3^rd^ and 4^th^ instar larvae and from pools of entire 1^st^ and 2^nd^ instar larvae. Extracted RNA was checked for integrity and size and then quantified using a Quant-IT RNA BR assay kit (Invitrogen). The single extracts were diluted to obtain a concentration of 100 ng/μl. Extraction was conducted in two different laboratories, and two pools of equal amounts of RNA were obtained, one for Russia and Camposampiero and one for Montello, for both adults and larvae. Each pool, consisting of a total of 5 µg of RNA, was stored in pure ethanol and shipped to Evrogen Labs Ltd., Moscow. The two adult and the two larval RNA pools were used for ds cDNA synthesis using the SMART approach [87]. SMART-prepared amplified cDNAs were pooled (ratio: ¾ of the Russia/Camposampiero pool and ¼ of the Montello pool) and then normalized using the DSN normalization method [88] to reduce overabundant transcripts. Normalization included cDNA denaturation/reassociation, a treatment by duplex-specific nuclease (DSN [89]) and the amplification of the normalized fraction by PCR.

### 454 pyrosequencing

Approximately 20 µg of normalized cDNA were used for the sequencing libraries construction (one for adults and one for larvae) at BMR Genomics, Padova, Italy, according to the described protocol [90]. The sequencing was performed on a half plate for each data set in a 454 GS-FLX titanium series pyrosequencer (Roche Applied Science).

### Preprocessing of 454 sequence reads

The raw reads from the two libraries were extracted from 454 SFF pyrograms through the open source alternative sff_extract 0.2.10. Sequence and qualities were tagged for the library of origin. We preprocessed the raw 454 sequence reads using the est_process module of the est2assembly 1.13 package [91], which performs sequencing adaptor removal, low complexity region masking, quality trimming, poly A/T detection and removal. We further cleaned the data sets from contaminants such as bacterial, viral, 18S RNA and *Solanum tuberosum* sequences using Deconseq [92] with the parameters: coverage ≥90% and identity ≥94%. These preprocessing steps with est2assembly and Deconseq were also performed on the publicly available midgut larval data set.

### Assembly of 454 sequence reads

We assembled the transcriptomic sequence reads from the three data sets (adult, full larval [FL] and midgut larval [ML]) with one assembly round using the MIRA 3.2 assembler [39] on “EST” and “accurate” usage mode. Settings adopted for this *de novo* assembly round were those defined by the 454 pyrosequencing technology (mira -job = denovo, est, accurate, 454 –notraceinfo). A summary of parameters and quality of this assembly is provided in **File S1**.

### Annotation of the transcriptomic dataset using homology searches

We annotated *L. decemlineata* transcriptome sequences by similarity search using BLASTX [59]. We used batch BLAST similarity searches for the entire transcriptome locally conducted against the non-redundant (nr) peptide database (downloaded in October 2010, including all non-redundant GenBank CDS translations + PDB + SwissProt + PIR+ PRF) with E-values<1e^−3^. We utilized the Blast2GO suite [41] for functional annotation of transcripts, applying the function to map GO terms to transcripts with BLAST hits generated from BLAST searches against nr. Only ontologies obtained from hits of E-values<1e^−3^, annotation cut-offs >25, and a GO weight >1 were used for the annotation.

The taxonomic classifications of annotated contigs were computed using MEGAN 4 [93] based on the absolute best BLAST hits after excluding contigs with multiple best BLAST records.

### Identification of stage specific transcripts

In order to identify transcripts that were uniquely expressed in either the larval or the adult stage, we used the same approach described by Vidotto *et al.* [94] to identify library specific contigs for transcriptome completeness estimation. First combined contigs were classified as being “adult”, “larva” or “common” on the bases of the reads composition. Larval contigs were further divided into FL and ML contigs, if originated from full larval and midgut larval datasets, respectively. Finally the common contigs were the fractions of those composed by reads from both libraries and then represented by transcripts considered to be expressed in the two developmental stages. We performed a bidirectional BLASTN [59] between the libraries specific contigs and those that align for more than 80% of their length, with e-values above 1e^−50^ were considered not library specific and then moved into the common fraction. The indirect subtraction was also performed to take into account contigs representing non overlapping fragments of the same transcript, which had not been assembled together. Library-specific contigs were searched for similarities, against the complete cDNA set of *T. castaneum*, stored in Ensembl Metazoa release-17 (Feb 2013), using TBLASTX [59]. The library-specific contigs with match against the same subjects, with e-values grater then 1e^−6^ and >80% query coverage, were considered belonging to the common fraction.

### Detection of molecular markers

Microsatellites were identified using Msatfinder version 2.0.9 software [95]. SNPs were predicted using the CLC Genomics *Workbench* 6 [75] with a criterion of at least 4 reads supporting either the consensus or variant within a minimum of 10 reads.

### miRNA detection

We scanned assembled sequences against all known miRNA sequences from miRbase Release 20 (June 2013) using BLAST suite [59] with E-values<1e^−3^.

### Search for genes of interest and phylogenetic analyses

We traced all genes of interest in the transcriptomic sequences using the BLAST suite [59] with E-values<1e^−3^. We aligned protein sequences from *D. melanogaster*, *T. castaneum* and those recovered from the *L. decemlineata* transcriptome for the specific protein superfamily under consideration using the MUSCLE alignment tool [96] with default settings. All phylogenetic trees were constructed using a Bayesian approach with 2 runs, a number of generations and protein model as specified in figures 4–6, and 25% burn-in-period in MrBayes 3.2 [97]. The best protein model for each gene was estimated using TOPALi V2.5 [98].

### Pfam Domain detection

We searched for all eukaryotic protein domains in the *L. decemlineata* transcriptome using the Pfam Domain Search function (which uses the HMMER software [99]) under the protein analysis module in the CLC Genomics Workbench 6.05 [70] with E-values<1e^−3^.

## Supporting Information

Figure S1 **Length distributions of** ***L. decemlineata*** **transcriptomic sequences.**
(PPTX)

Table S1 **List of contigs with nucleotide frequencies, length and percentage of GC content from transcriptomic sequences of** ***L. decemlineata*** **.** Contigs are named with suffix as per annotations provided by MIRA 3.2 assembler [39] as follow: c – “normal” contig; rep - these are contigs containing only repetitive areas; and s – singletons (contigs made up of a single read).
(XLS)

Table S2 **Gene Ontology (GO) annotation results of** ***L. decemlineata*** **transcriptomic sequences. A.** Biological Process. **B**. Molecular Function. **C**. cellular component.
(XLS)

Table S3 **Complete list of GO terms in the three BLAST2GO categories with enzyme code assignment.**
(XLSX)

Table S4 **List of KEGG pathways encompassing** ***L. decemlineata*** **sequences.**
(XLS)

Table S5 **List of putative miRNA genes from** ***L. decemlineata*** **454 transcriptomic sequences.**
(XLS)

Table S6 **List of** ***L. decemlineata*** **best hits with previously known diapause-specific genes obtained via suppressive subtractive hybridization.**
(XLSX)

Table S7 **List of putative genes involved in diapause in** ***D. montana*** **and their homologs in** ***L. decemlineata*** **transcriptome. The** table also shows the number of contigs identified as library specific for each gene. FL – full larva; ML – midgut-larva Red - genes not found in *L. decemlineata* assembly.
(XLSX)

Table S8 **List of transcripts with hits on genes known to be involved in resistance to Bt-Cry proteins.** The table also shows the number of contigs identified as library specific for each gene. FL – full larva; ML – midgut-larva
(XLSX)

Table S9 **Putative SNPs in** ***L. decemlineata*** **transcriptomic sequences.**
(XLS)

Table S10 **Putative microsatellite loci in** ***L. decemlineata*** **transcriptomic sequences.** (A) Contigs wise microsatelites. (B) Repeat classes.
(XLSX)

File S1 **A summary of parameters and quality of** ***L. decemlineata*** **transcriptome assembly.**
(DOC)
