# Supplementary figures and images for: Sequencing, De Novo Assembly and Annotation of the Colorado Potato Beetle, Leptinotarsa decemlineata, Transcriptome
Source: PLoS One. 2014 Jan 23;9(1):e86012. doi: 10.1371/journal.pone.0086012 (PMC3900453; doi:10.1371/journal.pone.0086012)

## Slide 1
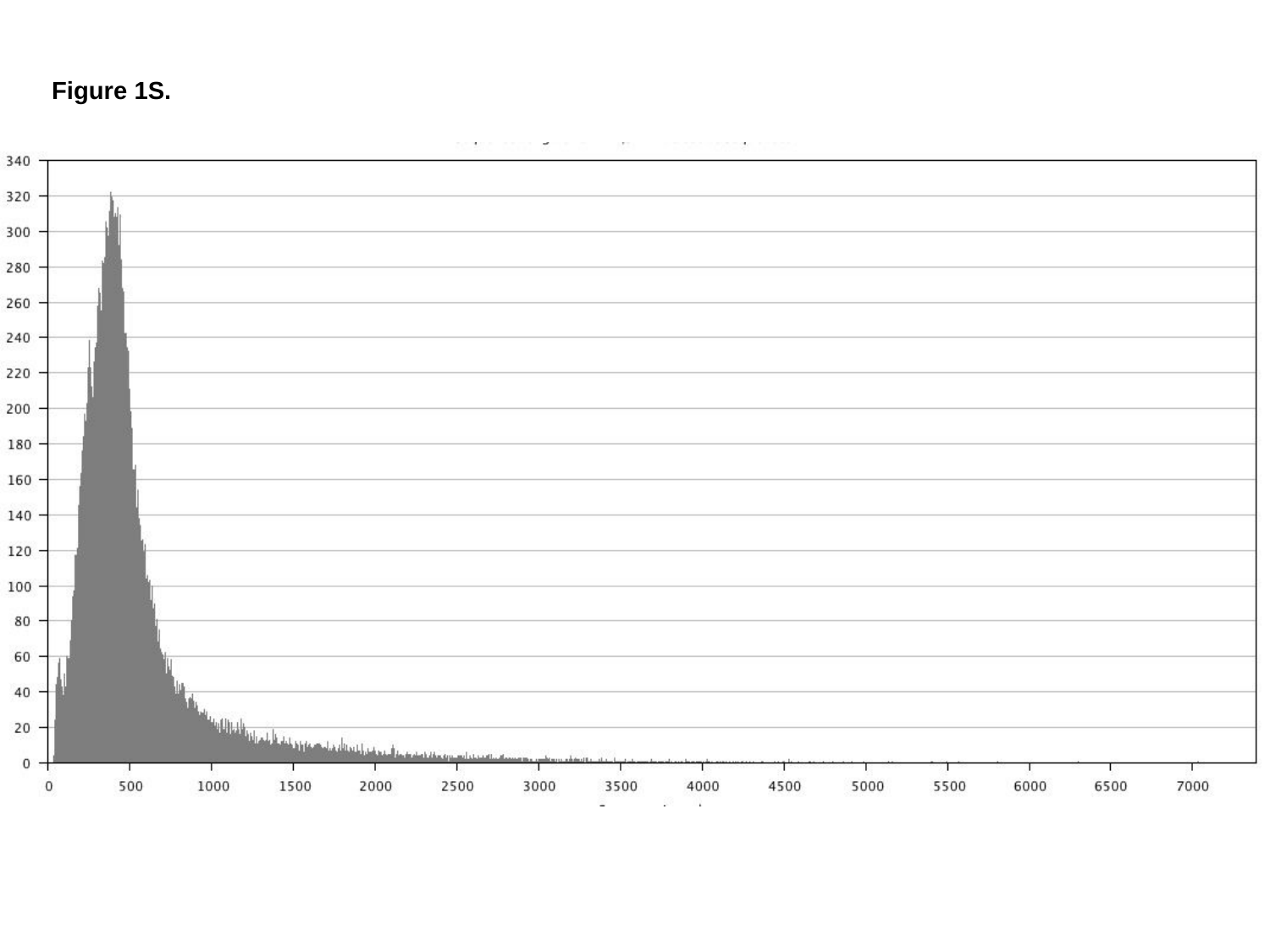

Figure 1S.

Supplement: Figure S1 — Length distributions of L. decemlineata transcriptomic sequences. (PPTX) [file pone.0086012.s001.pptx]
