# Supplementary material for: Sequencing, De Novo Assembly and Annotation of the Colorado Potato Beetle, Leptinotarsa decemlineata, Transcriptome
Source: PLoS One. 2014 Jan 23;9(1):e86012. doi: 10.1371/journal.pone.0086012 (PMC3900453; doi:10.1371/journal.pone.0086012)
Supplement: File S1 — A summary of parameters and quality of L. decemlineata transcriptome assembly. (DOC) [file pone.0086012.s012.doc]

------------------------------------------------

**Summary of Assembly parameters and quality**

------------------------------------------------

A) Mira callparameters

mira -job=denovo,est,accurate,454 -project=AcDNA COMMON_SETTINGS -GE:not=8 -LR:fo=no -SK:mnr=yes,mmhr=42 454_SETTINGS -CL:cpat=0:qc=0 -ED:ace=1 -OUT:sssip=yes -CO:fnicpst=yes -LR:mxti=yes -AS:mrl=40,bdq=10

B) **454 primer**

>primer_A_fwd

CGTATCGCCTCCCTCGCGCCATCAG

>primer_B_fwd

CTATGCGCCTTGCCAGCCCGCTCAG

C) **Quality assessment**

-------------------

Average consensus quality: 32

Consensus bases with IUPAC (IUPc): 0 (excellent)

Strong unresolved repeat positions (SRMc): 0 (excellent)

Weak unresolved repeat positions (WRMc): 0 (excellent)

Sequencing Type Mismatch Unsolved (STMU): 0 (excellent)

Contigs having only reads wo qual: 0 (excellent)

Contigs with reads wo qual values: 0 (excellent)

-------------------------------------------------------------------------------
